# Highly-accurate prediction of colorectal cancer through low abundance uncultivated genomes recovered using metagenomic co-assembly and binning approach

**DOI:** 10.1186/s12885-025-14787-5

**Published:** 2025-09-18

**Authors:** Po-Ting Lin, Yu-Wei Wu

**Affiliations:** 1 Department of Mechanical Engineering, National Taiwan University of Science and Technology, Taipei, 10607 Taiwan; 2 Intelligent Manufacturing Innovation Center, National Taiwan University of Science and Technology, Taipei, 10607 Taiwan; 3 Graduate Institute of Biomedical Informatics, College of Medical Science and Technology, Taipei Medical University, 250, Wuxing St., Sinyi District, Taipei, 11031 Taiwan; 4 Clinical Big Data Research Center, Taipei Medical University Hospital, Taipei, 11031 Taiwan; 5 TMU Research Center for Digestive Medicine, Taipei Medical University, Taipei, 11031 Taiwan

**Keywords:** Co-assembly, Binning, Colorectal cancer, Uncultivated species, Metagenome-assembled genome, Random forest feature importance

## Abstract

**Background:**

Recent microbiome studies have established the association between the composition of gut microbiota and various diseases. Since 16S ribosomal RNA sequencing may suffer from problems such as lower taxonomic resolution or limited sensitivity, more and more studies embraced whole-metagenome approach, which has the potential of sequencing everything in the target microbiome, to conduct microbial association analysis. However, species profiling, which is the most popular analysis technique for whole-metagenome analysis, cannot detect uncultivated species. Since uncultivated species may also be indispensable in the gut environments, it is crucial to identify those uncultivated species and evaluate their importance in discerning disease samples from healthy ones.

**Results:**

After conducting de novo co-assembly and genome binning procedures on two colorectal cancer (CRC) cohorts, in which one of them was from the Asian population while the other was from the Caucasian population, we identified that the Asian and Caucasian cohorts shared a significant amount of microbial species in their microbiota. In addition we found that low abundance genomes may be more important in classifying disease and healthy metagenomes. By sorting the genomes based on their random forest importance scores in differentiating disease and healthy samples and cumulatively evaluating the genome subsets in predicting CRC status, we identified dozens of “important” genomes for each of the cohorts that were able to predict CRC with very high accuracy (0.90 and 0.98 AUROC for the Asian and Caucasian cohorts respectively). Uncultivated species were also identified among the selected genomes, highlighting the importance of including the uncultivated species in order to build better disease prediction models and evaluate the roles of the uncultivated species in the disease formation or progression. Finally we found that the “important” species for both cohorts did not overlap with each other, hinting that the species highly associated with CRC disease may be different between the Eastern and Western populations.

**Conclusion:**

In this study we demonstrated the importance of recovering and analyzing low abundance uncultivated species to identify their associations with colorectal cancer. We hope this work shed new light on a more comprehensive understanding of how our gut microbes are correlated with diseases.

**Supplementary Information:**

The online version contains supplementary material available at 10.1186/s12885-025-14787-5.

## Introduction

It is already well-established that the collections of little microbes living inside our bodies can affect our health. Since the groundbreaking discovery that microbes were associated with and may lead to obesity [1, 2], numerous studies have been conducted to link diseases with the microbiota from our guts or other body parts (a few examples include type 2 diabetes [3], colorectal cancer [4, 5], cardiovascular disease [6], or inflammatory bowel disease [7], to name just a few). Neurological conditions or disorders such as Parkinson’s disease [8] or autism [9] were also linked to our gut microbiome, leading to the investigation of how gut microbes crosstalk with our brains (coined the gut-brain axis) [10]. Even lung and bone diseases such as chronic obstructive pulmonary disease (COPD) and osteoporosis/osteoarthritis/periodontitis were also found to be associated with the gut microbiota, forming the gut-lung [11] or gut-bone [12] axis. No wonder Feng et al. proposed that the gut microbiota “is an integral moderator in health and disease [13]”, as the microbes living in our gut seem to be associated with almost all diseases that we are aware of today.

In order to identify the links between gut microbiome and diseases, one would need to firstly compile the microbial composition of gut microbiome. Different sequencing and analysis techniques were employed to achieve this goal. The most commonly used sequencing techniques for studying gut microbiome include 16S ribosomal RNA amplicon sequencing and whole-metagenome sequencing, in which the former amplifies and extracts a portion of variable regions in the microbial 16S rRNA [14] while the latter aims to sequence every possible bit of the microbiota [15]. Since 16S rRNA amplicon sequencing may suffer from various disadvantages such as lower taxonomic resolution or limited sensitivity [16, 17], more and more recent studies employed whole-metagenome sequencing for its potential to sequence “everything” in the microbial community.

However, even though the whole-metagenome sequencing technique may have the ability to extract most of the genomic sequences, different analysis techniques may still yield different results. One of the most popular ways to compile the microbial composition of the whole-metagenome is through taxonomic profiling. In a nutshell, taxonomic profiling analysis is conducted by comparing the raw metagenomic reads against certain databases and quantifying the proportion of reads assigned to various taxa. Tools for taxonomic profiling have also been developed, including MetaPhlAn/MetaPhlAn2 [18, 19], Kraken/Kraken2 [20, 21], Centrifuge [22], Kaiju [23], and mOTUs/mOTUs2 [24, 25], to name just a few. Several large disease association studies have also employed profiling tools for extracting taxonomic information, including colorectal cancer [26–28], inflammatory bowel disease [29], autoimmune diseases [30], autism [31], or even COVID-19 [32], etc.

Another approach for conducting profiling and correlation analysis is through the MGWAS (metagenome-wide association study) approach proposed by Qin et al. in the type 2 diabetes metagenomic analysis [3]. Briefly the MGWAS approach was conducted by de novo assembling each individual metagenome, predicting genes from the assembled metagenomes, and assigning taxonomic, functional, and abundance information at the gene level for downstream statistical analysis. Several disease-microbiome association studies also employed MGWAS approach, including but not limited to hypertension [33], colorectal cancer [5], and rheumatoid arthritis [34].

One of the most noticeable characteristics of the aforementioned approaches (taxonomic profiling and MGWAS) is that they were all based on known taxonomic information, i.e., databases comprised of known species, genomes, or genes. Indeed comparing to existing databases allows very convenient extraction and compilation of species profiles to be associated with diseases; however these approaches may suffer from one drawback: they cannot identify unknown or uncultivated microbial species–coined “the microbial dark matter [35]”–that do not exist in databases. In other words, the profiling and MGWAS approaches are capable of finding known microbes that are associated with diseases; but they cannot say anything about the uncultivated ones.

Due to the plethora of uncultivated species identified in the human gut environments [36–40], studies have started to adopt sequence assembly-based methodologies to address the challenges of quantifying uncultivated organisms. One of the most popular approaches is through unsupervised genome binning or reconstruction from assembled metagenomes [41, 42]. Popular tools include MaxBin [43–45], MetaBAT [46, 47], CONCOCT [48], MyCC [49], DASTools [50], COCACOLA [51], MetaWrap [52], etc. have been developed for the genome binning purpose, and many studies such as integrated mouse gut metagenome catalog (iMGMC) [38], unified human gastrointestinal genome (UHGG) [36], or ruminant gastrointestinal tract microbiome [40] utilized binning tools for recovering uncultured candidate species from the sequenced metagenomes. The recovered genomes can then be analyzed to find their correlations with diseases [53], allowing the association of uncultivated candidate species with diseases or other phenotypes.

One of the potential drawbacks for the aforementioned large-scale genome recovery projects is that those studies recovered genomes from individual metagenomes instead of utilizing the power of multiple metagenomes at the same time. Studies have shown that co-assembly of metagenomes or single-cell genomes allows the recovery of higher quality genomes, and that the genomes with better assembly and binning qualities can be utilized for downstream analysis [54–58]. The co-assembly strategy may also allow the recovery of low-abundance genomes by increasing the sequence depth and improving assembly completeness [59], allowing more comprehensive genome comparisons across different metagenomic samples (Table 1).

**Table 1:** The distributions of healthy and disease samples in the Asian and Caucasian cohorts

Cohort	Sample number	CRC^a^ samples	Healthy samples	Reference
Asian	128	74	54	Yu 2017 [5]
Caucasian	109^b^	46	63	Feng 2015 [4]

^a^*CRC* colorectal cancer

^b^Advanced adenoma samples were excluded from the samples and were thus not included in the analysis. See Materials and Methods for details

Here we introduced the co-assembly analysis results of two colorectal cancer (CRC) cohorts, in which one was from the Asian population [5] while the other was from the Caucasian population [4]. By performing co-assembly on both cohorts and recovered metagenome-assembled genomes (MAGs), we demonstrated that low-abundance genomes may be more significantly associated with disease samples, and that very accurate CRC prediction can be achieved by a carefully selected MAG set. We also identified uncultivated species among the carefully selected MAGs, revealing the importance of taking uncultivated species into account in disease-association studies.

## Results

### Recovering genomes from the co-assembled metagenomes

**Fig. 1:**
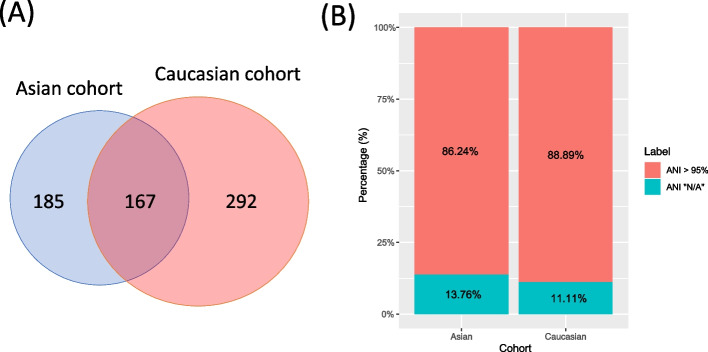
Taxonomic and average nucleotide identity (ANI) distribution of the genomes extracted from the Asian and Caucasian cohorts. **A** A Venn diagram showing the number of species overlapped between the two cohorts; **B** the proportion of genomes with ANI annotated as “N/A”, indicating that the ANI values of these genomes with the annotated species are below 95% and suggesting that these genomes may belong to uncultivated species

The metagenomic sequencing data of the Asian and Caucasian cohorts were obtained from two unrelated studies [4, 5]. The number of people in the case (that is, CRC) and control (healthy) groups of the two cohorts were as follows: the case and control group sizes of the Asian cohort were 74 and 54 while the group sizes of the Caucasian cohort were 46 63 respectively. Metagenome co-assembly were conducted de novo for each of the cohorts, yielding two co-assembled scaffold collections. The IDs of samples included in the analysis as well as the number of reads were Listed in Additional file 1: Table S1. After recovering MAGs from the scaffolds and keeping only genomes that satisfied at least medium quality requirement (completeness > 50%, contamination < 10%) as defined by MIMAG standard [60], totally 351 and 458 genomes were retrieved from the Asian and Caucasian cohorts respectively. GTDB-tk [61] was utilized to identify the most closely-related species along with average nucleotide identities (ANI) for the MAGs. After comparing the annotated species distribution of the MAGs for the two cohorts, we found that 167 MAGs shared the same annotation, as shown in Fig. 1(A). There were, however, 39 and 63 genomes annotated as “N/A” in the ANI field for the two cohorts. Since the GTDB-tk defined the lowest ANI score as 95% for confidently annotating the genomes, the genomes with “N/A” in their ANI fields may belong to uncultivated species since their ANI to the closest species is too low. As shown in Fig. 1(B), the proportion of potentially uncultivated genomes without ANI values were 13.76% and 11.11% for the Asian and Caucasian cohorts respectively. We also identified 15 uncultivated species present in both of the cohorts (i.e. genomes with the same taxonomic assignment and “N/A” in their ANI fields for both cohorts).

### Genome abundance distribution in disease and healthy samples

**Fig. 2:**
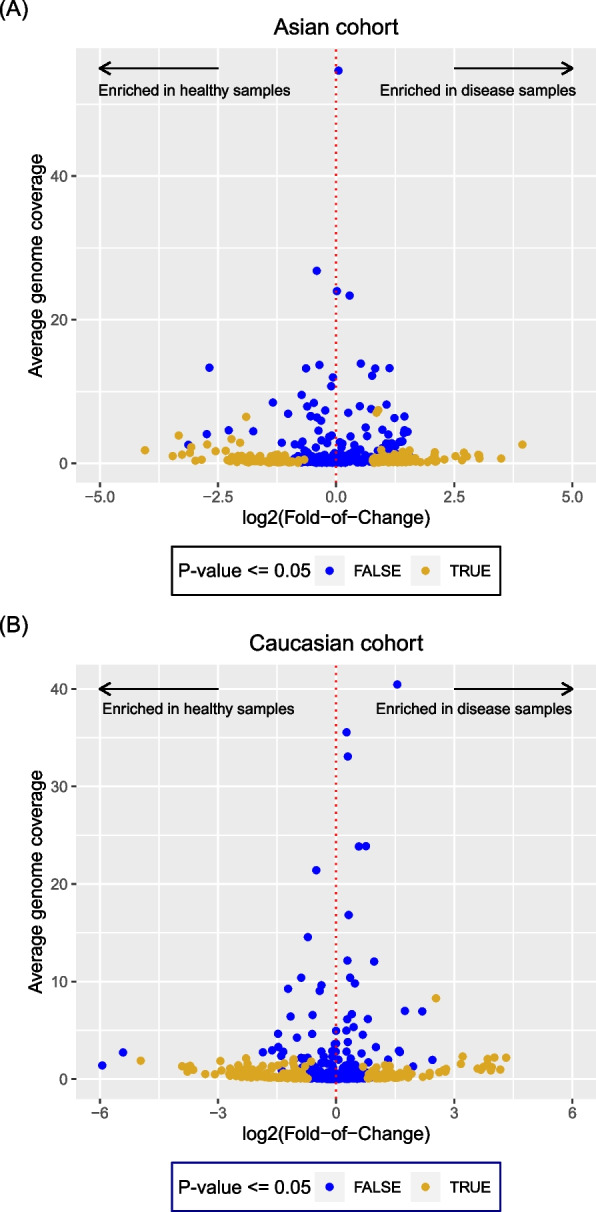
Scatter plots for the relationships of the averaged genome abundances and their log2-fold-of-change (see Materials and Methods) between disease and healthy samples. One dot represents one genome. The indicated genomes were extracted from (**A**) the Asian cohort, and (**B**) the Caucasian cohort. The significance of differential distribution between disease and healthy samples were also checked, in which the significantly distributed samples (i.e. *p* ≤ 0.05) were highlighted by golden color

By comparing the genome abundances between disease (colorectal cancer) and healthy samples, we found that low abundance genomes tend to be differentially-distributed between the two types of samples. The calculation of the log_2_(fold-of-change) (termed fold-of-change or FC hereafter) abundance differences between disease and heathy samples (see Materials and Methods; data available in Additional file 2: Table S2) revealed that genomes with low abundances were associated with high FC for both cohorts. As shown in Fig. 2(A) and (B), while low FC (located near the dotted red line in the figures) may be associated with both low and high genome abundances, high FC (far away from the dotted red line) were mostly associated with low genome abundances. The significance of differential distribution was also tested, in which most of the high FC genomes were significantly differentially distributed (*p* ≤ 0.05) between disease and healthy samples. In other words, we should look for low abundance genomes if we want to identify key species that were differentially-distributed between disease and healthy samples.

### Building prediction model for discerning disease and healthy samples

**Fig. 3:**
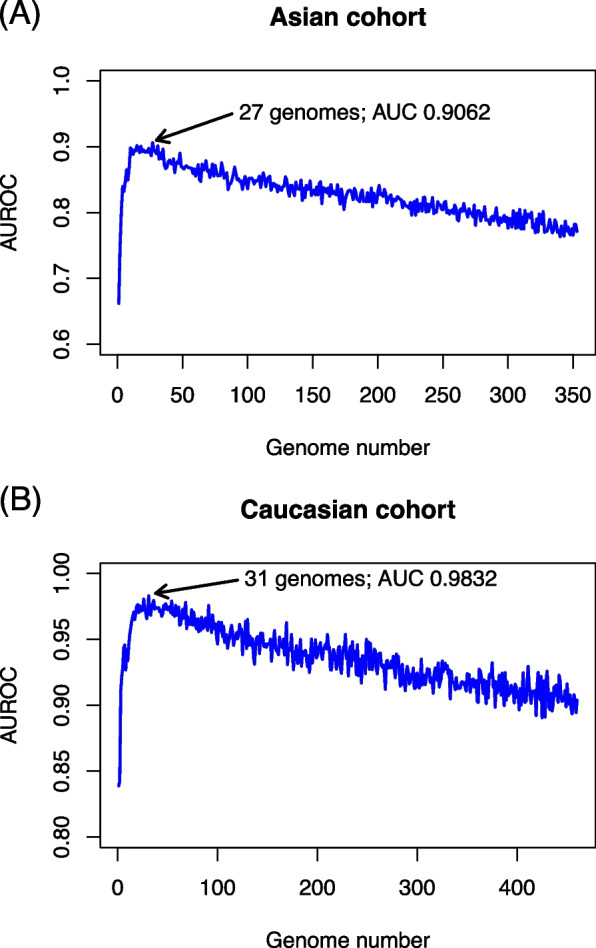
Line plots that show the number of cumulatively recruited genomes from high-to-low importance and the random forest prediction performances for the recruited genomes. Arrowheads indicate the highest prediction performance reached by the subset of cumulatively-extracted genomes. The performance metric is Area Under Receiver Operating Characteristics (AUROC); the evaluations were conducted using 10-fold cross-validation

Random forest models were built for both Asian and Caucasian cohorts using genome abundance data in order to extract genomes that may serve as better discriminators for disease and healthy samples. Since not all genomes were important or necessary for building the classification models, we adopted a random forest importance score-based cumulative approach for determining the feature (genome) set for building the best model (see Materials and Methods). As shown in Fig. 3, totally 27 and 31 genomes were picked from the genome collections of the two cohorts for building the best prediction models, and a repeated 10-fold cross-validation approach demonstrated that the prediction accuracy of the selected genomes reached 0.9062 and 0.9832 Area Under Receiver Operating Characteristic (AUROC, or AUC). In other words, for the two cohorts, the picked genomes were able to predict CRC status very accurately.

**Fig. 4:**
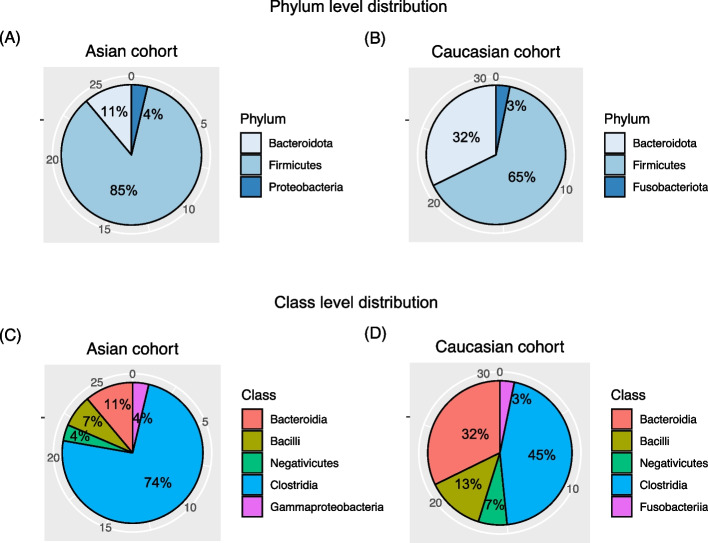
Pie charts demonstrating the phylum- and class-level taxonomic distributions of the extracted subsets of genomes that reach the best prediction performances. **A** Phylum-level distribution of the genomes selected from the Asian cohort; **B** phylum-level distribution of the genomes selected from the Caucasian cohort; **C** class-level distribution of the genomes selected from the Asian cohort; **D** class-level distribution of the genomes selected from the Caucasian cohort

The taxonomic distribution of the selected 27 and 31 genomes for the two cohorts showed that the majority of the genomes belonged to Firmicutes, and among the Firmicutes most of the genomes belonged to Clostridia. As shown in Fig. 4, the proportions of Clostridia among the selected genomes in the Asian and Caucasian cohorts were 74% and 45% respectively. The most “important” (i.e. with the highest importance score) genomes for the two cohorts, however, belonged to Bacteroidota, in which one was *Barnesiella intestinihomonis* (Asian cohort) while the other was *Prevotella copri* (Caucasian cohort). The detailed information of the selected genomes was available in (Additional file 3: Table S3). A comprehensive comparison between the shared genomes also showed that the 27 and 31 selected genomes of the two cohorts did not share species-level annotation, probably indicating that dietary and cultural differences between the Eastern and Western populations shaped the microbiota in different ways.

A closer investigation into the average abundances of the picked genomes revealed that the majority of the genomes were low abundance ones. Among the 27 and 31 selected genomes, 15 and 22 were lower than the mean abundances of the two cohorts (1.9066 and 1.447 for the Asian and Caucasian cohorts respectively). The percentages of low abundance genomes among the selected ones were 55.56% and 70.97%. This observation was consistent with the aforementioned fold-of-change/abundance analysis, which demonstrated that low abundance genomes tend to be differentially distributed between disease and healthy samples, as was shown in Fig. 2. The removal of low abundance genomes from the selected 27 and 31 genomes of the two cohorts resulted in 7% and 2% of prediction performances (0.8421 and 0.9628 AUROC respectively), indicating the necessity of including low abundance genomes into analysis.

Upon checking the ANI values of the selected genomes, we identified that two and four genomes were annotated as “N/A” in their ANI fields for the Asian and Caucasian cohorts, suggesting that these genomes belonged to uncultivated species. Even though those genomes were not the most “important” ones among the selected genomes, the existence of these uncultivated species among the selected genomes for building the best disease prediction models hinted that these genomes were also significantly correlated with CRC status and highlighted the necessity of extracting uncultivated species in disease-microbiota-association studies. We also attempted to remove the uncultivated genomes from the selected 27 and 31 genomes of the two cohorts, in which the prediction performances were decreased by 2% and 1.3% (0.886 and 0.970 respectively). The slight decrease again suggested that although the uncultivated genomes may not be the important genomes as was also indicated by the random forest importance score, the inclusion of these uncultivated genomes may still be beneficial to predicting disease status.

## Discussion

In this study we not only identified genomes that may belong to uncultivated species using metagenomic de novo co-assembly and unsupervised binning approach, but also found that low abundance species may be more closely related to the colorectal cancer disease status. In addition we also discovered that uncultivated species were among the most differentially-distributed species that can be used to build classification models. Since the majority of microbiome-disease association studies employed profiling methods that can identify only known species, this study showed that uncultivated species may also play important roles in predicting disease status. The co-assembly and binning approach employed in this study also allowed more convenient cross-sample comparison and highlighted how the uncultivated genomes can be incorporated into association and prediction studies.

Since the sequencing depth of every microbial organism is dependent on its abundance, the probably of retrieving sequencing reads from low abundance species is significantly lower than high abundance ones. As a result, unless the amount of total sequencing depth is very high, it is likely that low abundance species contributed too few reads to the metagenomes and thus cannot be assembled very well, and that the genome recovery process cannot find it at all due to poor assemblies. The metagenome co-assembly procedure can potentially mitigate this problem by piling up reads from low abundance species in order to increase their sequencing coverage and boost the likelihood of recovering their genomes. Despite the benefits, however, the co-assembly may still suffer from several pitfalls, including the requirement of intensive computation [62] and the possibility of mixing closely-related strains from the same species into the same genomic bin, resulting in ambiguous or fragmented assemblies [63]. The intensive need of computational resources, however, can also be partly mitigated by the use of suitable software packages. In this aspect we specifically chose MEGAHIT for three factors: its relatively faster speed [64], its comparably lower computation resources [65, 66], and its ability to generate better-than-average assemblies and capture strain-level variants [64]. Furthermore, in theory the memory or runtime of running co-assembly should be less than the sum of individual-assembly due to the shared k-mers present in multiple samples. We also argue that unless the sequencing depth of every individual metagenome is high enough to recover low abundance species, the co-assembly of metagenomes is still the most viable approach to obtain the genomes of low abundance species for analysis, as was demonstrated in this study.

By recovering genomes and annotating their most Likely taxonomic ranks from the two cohorts, in which one was an Asian cohort while the other was from a Caucasian population, we found that 167 species were shared between these two cohorts. The proportion of the shared species accounted for 47% and 36% for the Asian and Caucasian cohorts respectively. Since the gut microbiome composition was found to be convergent in the evolutionary process, in which chimpanzee and gorilla shared in average 53% of bacterial phylotypes [67] and that modern human and cercopithecines (a subfamily of Old World monkey) shared a significant amount of gut microbes, it is not surprising to identify common microbial species between the Eastern and Western people. The genome recovery process also allowed the extraction of uncultivated species, in which the uncultivated species accounted for 13.76% and 11.11% for the Asian and Caucasian cohorts. The successful mining of uncultivated species again highlighted the importance of recovering genomes from metagenomes in an unsupervised manner instead of relying on existing databases for profiling.

A statistical analysis on the differential distribution of microbial abundances between disease and healthy samples revealed that the genome abundance distribution differences were associated with low abundances. The plots shown in Fig. 2 clearly demonstrated that significantly high fold-of-change (FC) values were associated with low genome abundances across the cohorts, highlighting the importance of identifying low abundance species in the microbial populations. One of the possible reasons for the significant association of low abundance and high FC was that the low abundance species may be opportunistic pathogens that were capable of inducing diseases [68]. We note that low abundance species may appear at both high and low FC; hence it is recommended to get as many low abundance species as possible (potentially through the co-assembly process) in order to obtain genomes with abundance differences between disease and healthy samples.

By extracting the most “important” genomes from the two cohorts for disease classification purpose, we found that the selected genomes reached very high classification accuracy for the two cohorts, indicating the significant associations of the selected genomes with diseases. In addition we found that even though the majority of the selected genomes belonged to the Clostridia class, the most “important” genomes for the two cohorts were annotated as belonging to Bacteroidota phylum, in which one was *Barnesiella intestinihomonis* (Asian cohort) while the other was *Prevotella copri* (Caucasian cohort). It was not surprised to observe *Barnesiella intestinihomonis* species to be significantly enriched in CRC samples, as this species has been reported to promote the spread of cancer cells [69]. On the other hand, *Prevotella copri* was a controversial species [70], in which some studies reported it to be a beneficial microbe [71] while others found that *P. copri* may induce in vivo mucus degradation, resulting in the impairment of the mucosal barrier function and local inflammation [72]. Our data strongly suggested that in the Caucasian cohort, the identified *P. copri* species was significantly enriched in the disease samples, in which the average abundances of this species was 28.7 and 0.21 in CRC and healthy samples respectively. The huge differences strongly suggested that the extracted *P. copri* was the “bad guy” that may play a role in tumor development.

Despite the extraction of uncultivated species and the very high prediction accuracy achieved in this study, there are still a few aspects that we can continue improving. The first aspect was that after extracting microbial genomes from the co-assembly and binning process, we need to make sure that only genomes that met certain quality standards (medium quality in this case) were kept for downstream analysis, as both incomplete and/or highly-contaminated genomes may yield biased or inaccurate analysis results. The decontamination step, however, may also result in information loss by getting rid of genomes with uncharacterized roles in the microbiota. One possible solution is to combine the power of both known (profiling) and uncultivated (binning) information so that we can see a more comprehensive picture in microbiome-disease relationships. The implementation of this solution, however, is not straightforward in that the core ideas behind the two methods are very different such that integrating the results of these methods is challenging if not impossible. We will continue explore such possibilities in wielding the power of both types of methods together in order to extract more information from the microbiome data.

Another aspect that we can continue improving is the approach of selecting more crucial genomes/species sets for classifying disease and healthy samples. Also called “feature selection”, this type of approach focuses on selecting the minimal feature set that can achieve the best prediction performances through cross-validation procedure. There are many methods to conduct feature selection such as eXtreme Gradient BOOsting (XGBoost) [73], least absolute shrinking and selection operataion (LASSO) [74], genetic algorithm (GA) [75], support vector machine (SVM) [76], or other statistical methods such as analysis of variance (ANOVA) [77]. These and other feature selection methods may allow more concrete selection of highly-relevant species for downstream analysis. We hope that by selecting the optimal features we may achieve the goals of both constructing good prediction models and extracting the most informative species/genomes in discerning disease status. We also envision that the constructed disease-prediction models may have the potential to be translated into a non-invasive fecal metagenomics workflow for early detection of diseases in hospitals or healthcare centers.

## Conclusion

In this study we demonstrated that low abundance uncultivated species may also be highly associated with colorectal cancer disease status. We hope that by continually adding these uncultivated species into consideration we can achieve the goal of predicting diseases accurately from metagenomic data and understanding how diseases are associated with gut microbiome by analyzing the genomes of the uncultivated species.

## Materials and methods

### Co-assembly of the sequence data

The colorectal cancer shotgun metagenomic sequencing data from both Asian cohort and Caucasian cohort were downloaded from European Nucleotide Archive (ENA) accession PRJEB10878 [5] and ERP008729 [4]. The downloaded reads were first trimmed using Trimmomatic v0.39 (parameters: ILLUMINACLIP:TruSeq3-PE.fa:2:30:10 LEADING:12 TRAILING:12 SLIDINGWINDOW:4:15 MINLEN:36) [78] and then co-assembled for each of the cohorts using MEGAHIT v1.1.4–2-gd1998a1 with default parameters (–k-min 21 –k-max 141 –k-step 12) [79].

### Binning

The binning process was conducted using MaxBin 2.2.17 with default parameters (-min_contig_length 1000, -max-iteration 50) [44] for each of the co-assemblies. Sequencing reads of the metagenomic samples were input into MaxBin in order to calculate scaffold abundances, in which the forward and reverse-complement reads of each sample were concatenated into one file before inputting them into MaxBin, as instructed in [43]. After the binning process the genome abundances for each sample along with the recovered genomes were obtained from the MaxBin output files. Genome qualities were checked by checkM v1.1.3 using lineage_wf command with default parameters [80]. Only genomes with at least medium quality (at least 50% completeness and at most 10% contamination as instructed by MIMAG standard [60]) were kept for downstream analysis. The taxonomic assignments of the genomes and average nucleotide identity (ANI) to the closest genome were conducted using GTDB-tk v1.5.0 [61].

### Association analysis

The disease/healthy status were downloaded from Supplementary data of both studies [4, 5]. The samples designated as “adenoma”, which indicated the presence of advanced adenoma (dysplastic epithelium) that may or may not develop into carcinoma, were not included in the association study since only the Caucasian study consisted of adenoma samples. Genome abundances were extracted from the MaxBin results. The genome abundance calculation procedure of MaxBin was briefly described as follows. Firstly, the averaged sequencing coverage were estimated for all contigs, in which the sequencing coverage for contig *m* was calculated as follows.

$$coverage(m)=\frac{\sum_{r\in sequencing\ reads}\ count(nucleotides\ in\ r\ mapped\ to\ m)}{length(m)}$$

MaxBin utilized bowtie2 v2.2.3 [81] for reads mapping purpose. The abundance for any genome *G* can then be calculated as:

$$GenomeCov\left(G\right)=\frac{\sum_{contig\;m\in G}coverage(m)}{\sum_{contig\;m\in G}length(m)}$$

The log2(fold-of-change), defined as the difference between the averaged genome abundances of disease and healthy samples, were calculated for each genome. The formula for calculating the fold-of-change is as follows.

$$Disease(Abun{d}_{G})=\frac{1}{count(Disease)}\sum_{m\in Disease}abundance({G}_{m})$$

$$Healthy(Abun{d}_{G})=\frac{1}{count(Healthy)}\sum_{m\in Healthy}abundance({G}_{m})$$

$$\text{log}2(\text{Fold of change})={\text{log}}_{2}(\frac{Disease\left(Abun{d}_{G}\right)}{Healthy\left(Abun{d}_{G}\right)})$$

In which ${G}_{m}$ indicates a certain genome $G$ in sample $m$, $abundance({G}_{m})$ is the abundance of genome $G$ in sample $m$, and $Disease(Abun{d}_{G})$ and $Healthy(Abun{d}_{G})$ means the averaged abundance of genome $G$ in disease and healthy samples respectively. The significance of differential distribution were estimated using edgeR package v3.38.0 [82].

### Building prediction models for colorectal cancer prediction

A feature selection-based random forest prediction approach was adopted in building prediction models for both Asian and Caucasian cohorts. There were two steps in this approach. The first step was extracting the importance scores for the genomes in differentiating disease and healthy samples. The importance scores were calculated by building random forest classification models on the genome abundance values of both cohorts and extracting the feature importance values (mean decrease accuracy) for the genomes. The Scikit-learn Python package [83] was used for the random forest model implementation with the number of trees (parameter “n_estimator” in scikit-learn random forest package setting) set to 1,000 for the importance score extraction tasks.

After obtaining the importance scores, the second step was sorting the importance scores, in which high importance genomes were ranked higher, and adding these genomes, one-by-one based on the sorted order, into the feature set to build the prediction model. A 10-fold stratified cross-validation approach was conducted to evaluate the prediction performances of the selected feature set. To minimize the random effect the cross-validation procedure was executed three times for each of the feature set, and the evaluation results of all three runs were averaged to obtain more objective outcomes. The evaluation metric was Area Under Receiver Operating Characteristic (AUROC, or AUC). The feature (genome) sets with the best repeated cross-validation prediction performances were output as the best feature sets for both cohorts.

## Supplementary Information

Additional file 1: Table S1. List of metagenomic samples for the Asian and Caucasian cohorts. The sample status (case/control) and number off reads were also listed, in which paired-end reads were counted as one read.

Additional file 2: Table S2. The average genome coverage, the log_2_ fold-of-change, and the significances (i.e. *p*-value and false discovery rate) of the coverage differences calculated for the genomes recovered from the Asian and Caucasian cohorts.

Additional file 3: Table S3. Statistics of the selected 27 and 31 genome sets with the best prediction performances for the Asian and Caucasian cohorts.

## Data Availability

The analysis was conducted using public data from European Nucleotide Archive (ENA) accession PRJEB10878 and ERP008729.

## Abbreviations

CRC: Colorectal cancer
AUROC: Area Under Receiver Operating Characteristic
AUC: Area Under Receiver Operating Characteristic
COPD: Chronic Obstructive Pulmonary Disease
MGWAS: MetaGenome-Wide Association Study
LASSO: Least Absolute Shrinking and Selection Operation
GA: Genetic Algorithm
SVM: Support Vector Machine
ANOVA: Analysis of Variance
XGBoost: EXtreme Gradient Boosting
MIMAG: Minimum Information about a Metagenome-Assembled Genome
